# Is Virtual Reality Effective for Balance Recovery in Patients with Spinal Cord Injury? A Systematic Review and Meta-Analysis

**DOI:** 10.3390/jcm9092861

**Published:** 2020-09-04

**Authors:** Amaranta De Miguel-Rubio, M. Dolores Rubio, Alejandro Salazar, Jose A. Moral-Munoz, Francisco Requena, Rocio Camacho, David Lucena-Anton

**Affiliations:** 1 Department of Nursing, Pharmacology and Physiotherapy, University of Córdoba, 14004 Cordoba, Spain; z42mirua@uco.es; 2 Department of Cell Biology, Physiology and Immunology, University of Cordoba, 14007 Cordoba, Spain; ba1rulum@uco.es (M.D.R.); v02redof@uco.es (F.R.); m92caagr@uco.es (R.C.); 3 Department of Statistics and Operational Research, University of Cadiz, 11009 Cadiz, Spain; alejandro.salazar@uca.es; 4 Institute of Research and Innovation in Biomedical Sciences of the Province of Cadiz (INiBICA), University of Cadiz, 11009 Cadiz, Spain; 5 The Observatory of Pain, University of Cadiz, 11009 Cadiz, Spain; 6 Department of Nursing and Physiotherapy, University of Cadiz, 11009 Cadiz, Spain; david.lucena@uca.es

**Keywords:** virtual reality, spinal cord injuries, neurological rehabilitation, postural balance, physical therapy

## Abstract

Virtual reality (VR) is an emerging tool used in the neurological rehabilitation of patients with spinal cord injury (SCI), focused on recovering balance, mobility, and motor function, among other functional outcomes. The main objective of this study was to analyze the effectiveness of VR systems to recover balance in patients with SCI. The literature search was performed between October and December 2019 in the following databases: Embase, Web of Science, CINAHL, Scopus, Medline, Physiotherapy Evidence Database (PEDro), PubMed, and the Cochrane Central Register of Controlled Trials. The methodological quality of each study was assessed using the Spinal Cord Injury Rehabilitation Evidence (SCIRE) system and the PEDro scale, while the risk of bias was analyzed by the Cochrane Collaboration’s tool. A total of 12 studies, involving 188 participants, were included in the systematic review, of which two were included in the meta-analysis. Statistical analysis showed favorable results for balance measured by the modified Functional Reach Test (standardized mean difference (SMD) = 3.42; 95% confidence interval: 2.54 to 4.29) and by the t-shirt test (SMD= −2.29; 95% confidence interval: −3.00 to −1.59). The results showed that VR interventions provided potential benefits, in addition to conventional physical therapy, to recover balance in patients with SCI.

## 1. Introduction

Spinal cord injury (SCI) involves an alteration of the spinal cord that causes a disorder or loss of proprioception, mobility, or autonomous function [1,2]. The neurological rehabilitation of patients with SCI is focused on recovering functional performance, mobility, and balance, among others [3]. To achieve balance, the coordination and integration of different body system are needed [4]. Balance is required to perform most of the activities of daily living, and balance impairments could provoke mobility, posture, and gait disturbances [5].

One of the emerging rehabilitation tools in recent years is the application of virtual reality (VR)-based technologies [6]. VR is a therapeutic option for rehabilitation in neurological disorders and the use of this technology has increased in recent years. VR comprises two types of systems according to the immersion level: (i) semi-immersive or non-immersive systems, and (ii) immersive systems. Semi-immersive and non-immersive systems use a screen to display the environment with a low level of immersion. Commercial videogame consoles are included in this type of VR. Immersive systems offer full integration of the user into the virtual environment and these systems can incorporate other devices (e.g. gloves, exoskeletons, etc.) to provide sensory inputs to the patient. VR caves, large-screen projections, and head-mounted displays are considered as immersive VR systems [7].

Most studies analyzed the effects of VR interventions in patients with stroke [6,8,9,10], cerebral palsy [11,12], Parkinson’s disease [13,14], and multiple sclerosis [15,16,17]. Several studies have analyzed the application of this technique, compared to conventional physical therapy (CPT), in patients with SCI [18,19,20,21,22,23,24]. VR-based therapy offers the opportunity to practice sports, recreational or functional activities without any risk; activities whose practice could be dangerous in real scenarios [25]. Virtual reality-based interventions have been proposed as a complementary approach to conventional treatment and can increase the intensity of repetitive practice, keeping motivation in subjects [18] and acting by providing multisensory feedback in an immersive environment with high ecological validity [25,26,27]. These five characteristics (intensity, repetition, motivation, feedback, and specificity of the task) could stimulate motor learning processes and thus produce effects on balance.

Three recent reviews have been conducted analyzing the effects of VR in patients with SCI. Yeo et al. [28] performed a structured review aiming to know the evidence of using VR to improve the mobility of patients with SCI. They included seven case series and two randomized controlled trials and concluded that VR is effective to improve balance and posture. A systematic review conducted by Araújo et al. [29], aiming to evaluate the benefits of VR in patients with SCI, included 25 studies; 12 of them used a pre-post design without a control group, 13 were controlled in a parallel or crossover design, and only 11 studies used randomization between groups. They suggested that VR interventions could provide benefits on balance and motor function recovery. Finally, Abou et al. [30] conducted a systematic review and meta-analysis aiming to know the effect of VR on gait and balance among patients with SCI. It included 10 trials, 3 randomized clinical trials, and 7 used pre-post design without a control group. They concluded that this therapy has beneficial effects, enhancing sitting and standing balance, and showed gait improvements. Nevertheless, a systematic review and meta-analysis analyzing the potential benefits of using VR systems specifically to improve balance in patients with SCI is still needed. Based on this background, we hypothesize that VR interventions could be an additional therapy, improving the effects of the rehabilitation process on balance. Therefore, the objective of this review and meta-analysis was to analyze the effectiveness of VR systems to recover balance in patients with SCI.

## 2. Materials and Methods

### 2.1. Search Strategy

The PRISMA (Preferred Reporting Items for Systematic Reviews and Meta-Analyses) [31] guidelines were followed to perform this systematic review. The search protocol was registered in the PROSPERO database of prospectively registered systematic reviews (CRD 42018093855). The literature search was performed between October and December 2019 in the following electronic databases: Embase, Web of Science, CINAHL (Cumulative Index to Nursing and Allied Health Literature), Scopus, Medline, Physiotherapy Evidence Database (PEDro), PubMed, and the Cochrane Central Register of Controlled Trials. The following descriptor terms combined with Boolean operators were employed: (“spinal cord injuries” OR “spinal cord injury” OR “quadriplegia” OR “tetraplegia” OR “paraplegia”) AND (“virtual reality exposure therapy” OR “virtual reality” OR “augmented reality” OR “virtual systems” OR "videogames" OR “video games” OR “exergames” OR “exergaming” OR “commercial games” OR “play-based therapy”). In the PubMed database, Medical Subjects Headings (MeSH) descriptors were used: “spinal cord injuries,” “virtual reality exposure therapy,” “virtual reality,” and “video games.” In addition, we checked the reference lists of relevant articles to identify further published trials. No language and date filters were applied.

### 2.2. Selection Criteria

The PICO (Population, Intervention, Comparison, Outcomes) model was employed to define the selection criteria, where the Population was adults diagnosed with SCI, the Intervention was VR therapy (immersive, semi-immersive, and non-immersive systems), the Comparison was adults with and without SCI undertaking CPT, and the Outcomes were specifically related to balance (sitting and standing). Clinical trials were considered as study type. The following criteria were taken into account to exclude articles: participants were people with SCI and other pathologies, but the outcome data were not provided for each specific population. Furthermore, single-case studies were excluded, since they do not add enough evidence to our analysis and are lacking means and standard deviations, which are needed to calculate appropriate statistics.

Regarding the comparison, it is important to remark that we considered as CPT any standardized exercise used into the physical therapy program aiming to enhance sitting or standing balance, as well as any exercise focused on strengthening and/or stretching musculature [30], as defined by the World Confederation of Physical Therapy (WCPT) [32].

### 2.3. Study Selection Process and Data Extraction

The first action, once the literature search was carried out, was to exclude duplicated articles. Then, titles and abstracts were reviewed, and we excluded those articles that did not meet the established inclusion criteria. The remaining articles were rigorously analyzed to obtain the articles included in the systematic review. Two reviewers (A.M.R. and M.D.R.L.) took part independently in the study selection process, review, and systematic data extraction. A third reviewer (D.L.A.) participated in the final decision in cases of doubt. The data extracted from the studies were: (1) author and date of publication; (2) level of evidence according to the study design; (3) number and age of participants, levels of injury and mean time post-onset; (4) characteristics of the interventions (intervention types in each group, outcome measures, measuring instrument) and results.

### 2.4. Assessment of the Risk of Bias and Methodological Quality of the Studies Included in the Review

The Cochrane Collaboration’s tool [33] was used to analyze the risk of bias. It was developed by the Review Manager 5.3 software. This tool includes an evaluation of different items in terms of risk of bias. The studies are categorized as: “unclear risk,” “low risk,” and “high risk.” Two reviewers conducted the risk of bias assessment. In cases of doubt, a third assessor took part in the final decision.

The methodological quality of each study was assessed using the Spinal Cord Injury Rehabilitation Evidence (SCIRE) system and the PEDro scale. The SCIRE system uses different categories to analyze the research design and methodological quality, grading from level 1 (highest quality) to 5 (lowest quality) [34]. In addition, the methodological quality of the randomized controlled trials was assessed by the PEDro [35] scale. This scale comprises different items related to the domains of selection, performance, detection, information, and attribution bases. A higher score shows a higher methodological quality. PEDro scores of six or higher are considered as a high level of methodological quality (6–8: good; 9–10: excellent), and scores of five or less are considered as low level of methodological quality (4–5: acceptable; <4: poor) [36].

### 2.5. Statistical Analysis

The measuring instrument used in each study determined the subgroups for the meta-analyses. When a study used more than one instrument, it was included in more than one subgroup. All the groups compared CPT vs. VR. The standardized mean differences (post-pre intervention) were the measures of the effect size between the groups. Results are presented with 95% confidence intervals, and the significance level was set at *p* < 0.05. 

The heterogeneity was analyzed by means of the chi-square test and the I2 statistic. Subsequently, random-effects models or fixed-effects models were used where heterogeneity or homogeneity were observed, respectively.

The software Review Manager (RevMan) 5.3 (The Cochrane Collaboration, The Nordic Cochrane Centre, Copenhagen, Denmark) was used. The results are presented tabulated and in the forest plots.

## 3. Results

**Figure 1:**
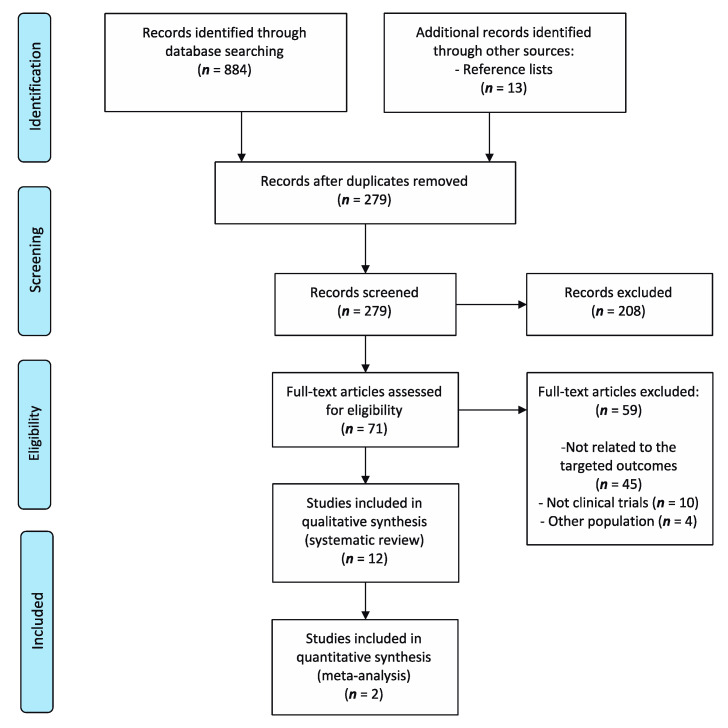
Information flow diagram of the selection process of the systematic review and meta-analysis.

As shown in Figure 1, the literature search retrieved a total of 884 records. A total of 12 studies were included in the systematic review, of which two were included in the meta-analysis for statistical comparison.

### 3.1. Data Extraction.

A total of 188 subjects (Comparison group (CG), *n* = 57; Intervention group (IG), *n* = 131) took part in the different studies. The highest number of participants was achieved by D’Addio et al. [22] (*n* = 30) and Khurana et al. [37] (*n* = 30). In contrast, only two subjects participated in the study by Roopchand-Martin and Bateman [38]. The average age of the participants ranged from 19 [38] to 60 [39]. Concerning the neurological level of injury, most studies included participants injured at cervical or thoracic levels. According to the American Spinal Injury Association Impairment Scale (ASIA), most studies included participants with C–D levels. The main characteristics of the participants are shown in Table 1.

Regarding the intervention protocols, all the studies analyzed the effects of VR interventions through different technological devices compared to CPT. In terms of VR systems, most studies used the Nintendo Wii videogame console [22,38,44,45], video-capture systems [46,47], and VR-augmented therapy [39,41,42]. Another study [40] employed a VR system similar to the Nintendo Wii, based on force plates, and, finally, the last study [37] used the PlayStation 2 videogame console.

Concerning the duration and intensity of the protocols, the longest total duration of intervention was achieved by D’Addio et al. [22] with a total of 12 weeks. The longest session duration (60 min) was achieved by Sayenko et al. [40], Wall et al. [45] and van Dijsseldonk et al. [47], and the highest program intensity was carried out by Khurana et al. [37] (five times a week). 

Regarding the different deficits treated, all the studies focused on their interventions to recover balance in patients with SCI. Specifically, four studies [37,38,43,44] analyzed the effects of VR interventions on sitting balance, while the remaining studies [22,39,40,41,42,45,46,47] focused on their interventions for recovering standing balance. In addition, most studies analyzed the effects of VR therapy in gait [39,41,42,45,47], and functional performance [22,37,39,41,42]. It should be highlighted that all the studies got positive results on balance recovery for VR interventions. Table 2 shows the main characteristics of the different interventions performed by the different studies.

### 3.2. Assessment of the Risk of Bias and Methodological Quality of the Studies Included in the Review

**Figure 2:**
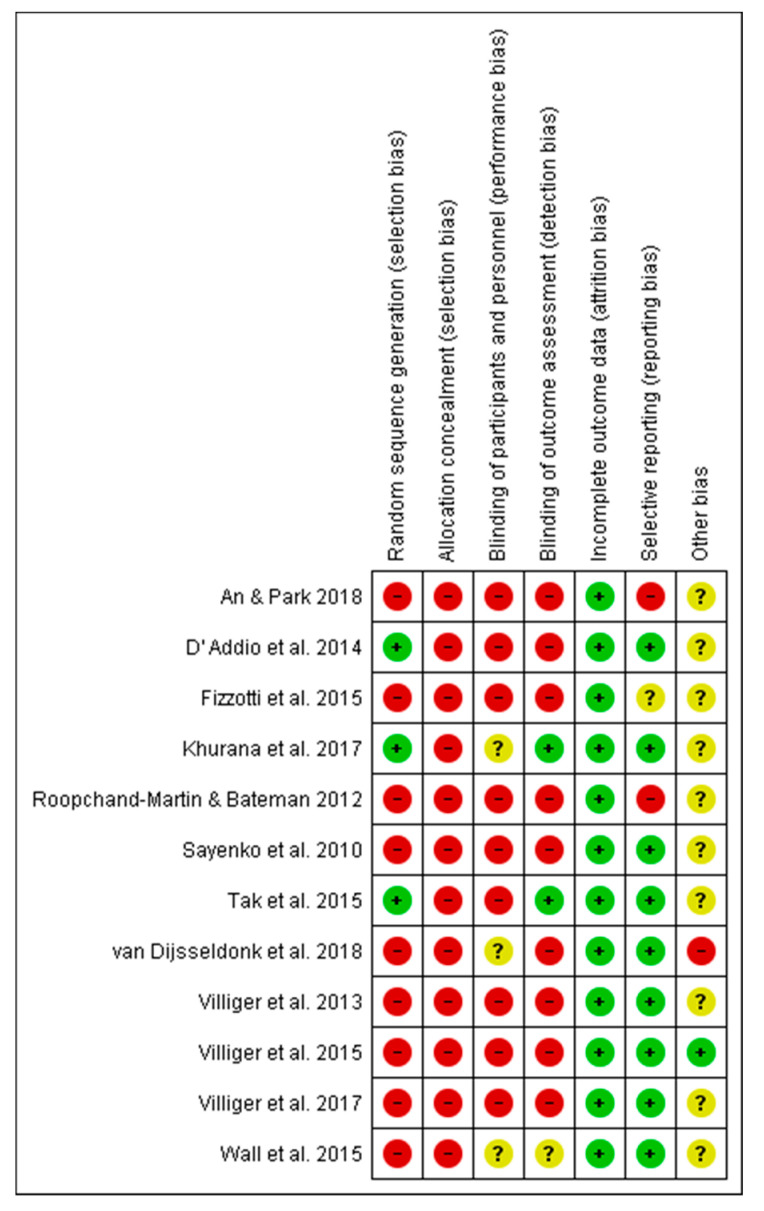
Risk of bias of the studies included in the systematic review.

**Figure 3:**
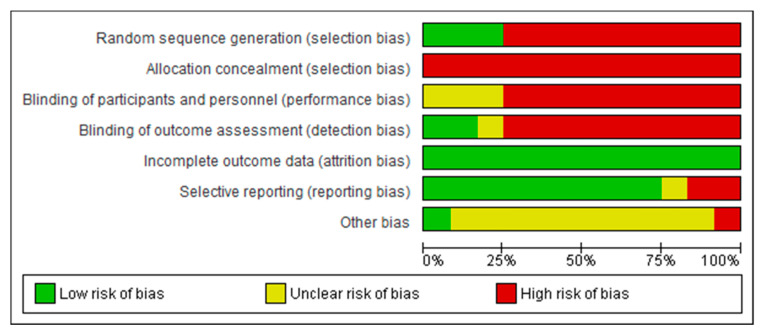
Overall risk of bias. Each category is presented by percentages.

Concerning the risk of bias of the studies included in this systematic review, the studies carried out by Khurana et al. [37], and Tak et al. [44] presented the lowest risk of bias. Conversely, the studies conducted by An and Park [46] and Roopchand-Martin and Bateman [38] presented the highest risk of bias. Furthermore, concerning the risk of bias among the studies analyzed, the lowest biases were found in the incomplete outcome data (0%) and the selective reporting (25%), while the highest value (100%) was found in the allocation concealment. The results are shown in Figure 2 and Figure 3.

The different scores obtained in the SCIRE and PEDro scales are shown in Table 1. Three studies were randomized controlled trials [22,37,44], while nine studies were cross-sectional and case-series studies. The methodological quality of the randomized controlled trials included in this review was generally good (average total PEDro score = 6.3, range 4–8). Two [37,44] of them had a high methodological quality, scoring equal to or higher than six points, as shown in Table 3. In addition, the other studies obtained a level four of evidence according to the SCIRE criteria.

### 3.3. Study Groups Included in the Meta-Analysis

Two tests were used by the studies to analyze the balance differences: the modified Functional Reach Test and the t-shirt test. Only two studies measuring sitting balance were included in the meta-analysis. Both Khurana et al. [37] and Tak et al. [44] used commercial videogame consoles to deliver balance training. All participants received CPT during the intervention program, although Khurana et al. [37] specified that the comparison group performed specific balance training exercises apart from the CPT, and Tak et al. [44] also included balance training in the comparison group. Furthermore, this kind of intervention is considered as part of the clinical CPT program [30,32]. Despite the fact that the controlled trial by D’Addio et al. [22] analyzed the effects of VR interventions on balance recovery, it could not be included in the meta-analysis due to the different tests used to analyze the effects.

**Figure 4:**
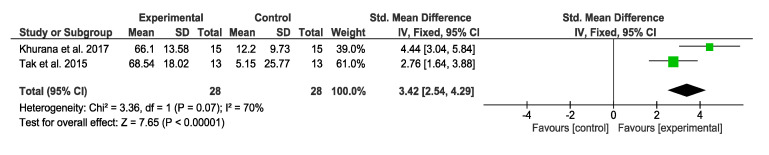
Forest plot for balance measured by the modified Functional Reach Test. Green block indicates the weight assigned to the study and the horizontal line depicts the confidence interval. Black rhombus shows the overall result.

Concerning the modified Functional Reach Test, two studies [37,44] analyzed the effects through this test, and both studies obtained significant results for VR therapy. The overall result of this meta-analysis was favorable, as shown in Figure 4.

**Figure 5:**
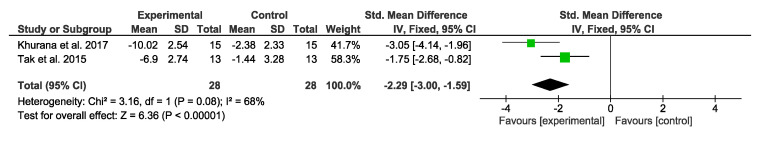
Forest plot for balance measured by the t-shirt test. Green block indicates the weight assigned to the study and the horizontal line depicts the confidence interval. Black rhombus shows the overall result.

Regarding the results obtained in the t-shirt test, the same two studies [37,44] analyzed the effects of VR interventions through this test. Significant results for VR interventions were obtained in both studies. The overall result of this meta-analysis was favorable, as shown in Figure 5.

## 4. Discussion

The present research aimed to analyze through meta-analysis the effectiveness of VR interventions on balance recovery in patients with SCI, compared to CPT. A total of 12 articles were reviewed and two were included in the meta-analysis. A total of 188 subjects took part in the different studies. It must be emphasized that all the studies reported significant effects on balance recovery for VR interventions compared to CPT, so we can conclude that VR interventions provided potential benefits in addition to CPT to recover balance in patients with SCI.

The present findings are supported by the structured review carried out by Yeo et al. [28], which analyzed the effectiveness of VR therapy for improvement of mobility and showed potential benefits on balance. Furthermore, our results match with those obtained by de Araújo et al. [29] and Abou et al. [30], who suggested that VR-based rehabilitation may lead to positive effects on balance in patients with SCI. Conversely, the present findings do not match with those obtained by our previous meta-analysis [3], performed to assess the effects of VR interventions on functional performance in patients with SCI. However, it should be noted that the only studies [22,37] that provided benefits on functional performance are also the only studies that obtained significant results on balance. In the present review, all the studies [22,37,39,41,42] that analyzed the effects of VR interventions on functional performance and balance, got significant results in both outcomes, so we can reinforce our hypothesis that the significant results obtained in the functional performance are influenced by the significant result obtained in balance, since functional abilities and balance recovery are positively correlated [44]. In addition, we can suggest that, following the International Classification of Functioning, Disability and Health (ICF) [48], balance weakness could affect the functional performance and vice versa.

Concerning the VR devices used, despite all the studies performing VR interventions through semi-immersive or non-immersive systems, where a computer or videogame console displays the virtual environments through screens [49], all of them got positive effects on balance recovery. Regarding the participant’s characteristics, there is heterogeneity in terms of the injury severity (measured by ASIA scale) and the levels of injury. Therefore, we cannot assure that these factors could affect the results obtained.

It should be emphasized that the positive results obtained in the present review could have an impact in clinical neurorehabilitation, since patients with SCI usually present severe limitations on their participation and performance of the activities of daily living [1,20]. Furthermore, the inclusion of VR interventions in clinical practice could generate more patient motivation and treatment adherence [24], performance of different activities in virtual safe environments [49], and provision of feedback and task-oriented training [6].

Although relevant findings are shown in this study, we should remark on some limitations. The limited number of studies analyzed implies that the results must be taken with caution. Furthermore, the small sample sizes and the different injury levels of the patients included in the studies could influence the statistical analysis, misrepresenting the results obtained. Thus, the use of large sample sizes and the inclusion of a proper number of subjects in stratified groups, to know which factors of the participant’s characteristics could influence the results, are needed. Nevertheless, most studies include convenience samples, since these patients are usually treated in neurologic institutions or centers. It is difficult to get large sample sizes in this clinical setting, increasing the subject selection bias [50]. Furthermore, other factors affecting the results could be the heterogeneous protocols used, the different program and session durations, and the different CPT protocols used. Therefore, randomized controlled trials with higher methodological quality using larger sample sizes are needed. It is also important to unify VR intervention protocols, identifying the key aspects of VR interventions that could have a greater impact in achieving the intended effects on balance recovery after SCI. 

## 5. Conclusions

The results of this systematic review and meta-analysis showed potential benefits using VR interventions in addition to CPT to recover balance in patients with SCI. Nonetheless, these findings should be taken with caution, since the results obtained are based on poorly designed studies, providing weak evidence of the treatment effectiveness. 

In view of our conclusion, we encourage researchers to conduct randomized clinical trials with high methodological quality and adequate sample sizes. Furthermore, it is necessary to unify VR intervention protocols to provide further evidence of the use of VR intervention in neurological rehabilitation focusing on improving the balance, and consequently the quality of life of patients with SCI. 

## Figures and Tables

**Table 1:** Main characteristics of the participants in each study.

Study	Participants (*n*)	Age (Mean ± SD)	ASIA Grade	Level of Injury	Time after Onset Injury (Months)
Sayenko et al. 2010 [40]	*N* = 6	42 (27–62)	C–D	C4, T10–T12	9.17
Roopchand-Martin and Bateman. 2012 [38]	*N* = 2	19, 47	A	T4 and T12	7.5
Villiger et al. 2013 [41]	*N* = 14	52.7 ± 14.9	C–D	C4-C8, T11-T12	12–240
D’Addio et al. 2014 [22]	*N* = 30 CG: 15, IG: 15	43.0 ± 18.7	CG: C–D IG: C–D	ND	ND
Villiger et al.2015 [42]	*N* = 23 CG: 14, IG: 9	CG: 47.1 ± 14.4 IG: 55.1 ± 15.8	IG: C–D	IG: C4-C8, T12	IG: 12–60
Fizzoti et al. 2015 [43]	*N* = 15	37 (19–66)	A–C	ND	ND
Tak et al. 2015 [44]	*N* = 26 CG: 13, IG: 13	CG: 43.1 ± 11.23 IG: 49.5 ± 8.25	CG: A–B IG: A–B	CG: Cervical/Thoracic IG: Cervical/Thoracic	CG: 22.4 IG: 21.7
Wall et al. 2015 [45]	*N* = 5	58.6 (54–60)	D	C4–C6, L1	7.6
Khurana et al. 2017 [37]	*N* = 30 CG: 15, IG: 15	CG: 29.8 ± 7.32 IG: 29.4 ± 7.48	CG: A–B IG: A–B	CG: T6–T12 IG: T6–T12	CG: 2.6 IG: 3
Villiger et al. 2017 [39]	*N* = 12	60.0 ± 10.2	C–D	C4–C7, T4–T12, L3	12–204
An and Park 2018 [46]	*N* = 10	44.2 ± 8.66	C–D	C2–C7, T1	13–25
van Dijsseldonk et al. 2018 [47]	*N* = 15	59.0 ± 12.0	C–D	ND	42–48

ASIA: American Spinal Injury Association Impairment Scale; CG: Comparison group; IG: Intervention group; ND: Not described. Note: Detailed information divided into study groups was added when available.

**Table 2:** Main characteristics of the studies included in the systematic review.

Study	Scire/Pedro Scores	Group Interventions	Intensity	Session Duration	Intervention Duration	Outcome	Measuring Instrument	Results
Sayenko et al. 2010 [40]	SCIRE: Level 4	IG: VR Games + force plate	3 times/week	60 min	4 weeks	-Static and dynamic standing balance	Center of pressure (force plate)	Significant results were found during standing in game performance and training-irrelevant tasks
Roopchand-Martin and Bateman. 2012 [38]	SCIRE: Level 4	IG: Nintendo Wii	2 times/week	45	6 weeks	-Static sitting balance	mFRT	Both patients improved their balance ability
Villiger et al. 2013 [41]	SCIRE: Level 4	IG: VR-augmented training	4–5 times/week	45 min	4 weeks	-Standing balance -Gait -Mobility -Neuropathic pain -Motor function -Functional performance	10mWT, BBS, LEMS, SCIM, WISCI II, pain intensity and unpleasantness, PGIC	Significant differences were found in gait(*p* ≤ 0.001), balance (*p* ≤ 0.002), motor function (*p* ≤ 0.002), functional performance: SCIM (*p* ≤ 0.004), WISCI II (*p* ≤ 0.004), and neuropathic pain (*p* ≤ 0.008), PGIC(*p* ≤ 0.016)
D’Addio et al. 2014 [22]	SCIRE: Level 21PEDro: 6	CG: Patients with SCI: Conventional physical therapy IG: Patients with SCI. Nintendo Wii	3 times/week	ND	12 weeks	-Standing balance -Posture -Functional performance	BBS, Romberg, posturographic analysis, SCIM	Significant results between groups were found in all parameters: BBS (*p* = 0.02); Romberg (*p* = 0.03); posturography (*p* = 0.03 and *p* = 0.04); SCIM (*p* = 0.02)
Villiger et al.2015 [42]	SCIRE: Level 4	CG: Healthy subjects. Intense VR-augmented training IG: Patients with SCI. Intense VR-augmented training	4–5 times/week	45 min	4 weeks	-Standing balance -Gait -Motor function -Functional performance	10mWT, BBS, LEMS, SCIM	Significant differences were found in patients with SCI: gait (*p* ≤ 0.001), balance (*p* ≤ 0.001), motor function (*p* ≤ 0.001), and functional performance (*p* ≤ 0.001).
Fizzoti et al. 2015 [43]	SCIRE: Level 4	IG: Tablet-based VR system	2–3 times/week	ND	3–12 weeks	-Sitting balance (trunk postural control)	Trunk Recovery Scale	Significant results were found in trunk postural control (*p* = 0.013)
Tak et al. 2015 [44]	SCIRE: Level 1 PEDro: 76	CG: Conventional physical therapy, IG: Nintendo Wii	3 times/week	30 min	6 weeks	-Static sitting balance (postural sway distance, postural sway velocity) -Dynamic sitting balance	Force plate, mFRT, t-shirt test	Significant results between groups were found in static and dynamic balance: anterior-posterior and total postural sway distance (*p* < 0.05); anterior-posterior and total postural sway velocity (*p* < 0.05); left, front and right mFRT (*p* < 0.05); the T-shirt test (*p* < 0.05)
Wall et al. 2015 [45]	SCIRE: Level 4	IG: Nintendo Wii	2 times/week	60 min	7 weeks	-Standing balance -Gait speed -Functional mobility	10mWT, TUG, Forward and lateral FRT	Significant results were found in gait speed (*p* = 0.001) and forward FRT (*p* < 0.001), and lateral FRT (*p* = 0.001)
Khurana et al. 2017 [37]	SCIRE: Level 1 PEDro: 8	CG: Conventional physical therapy focused on balance training IG: Sony Play Station 2 + Eye Toy	5 times/week	45 min	3 weeks	-Sitting balance -Functional performance	mFRT, t-shirt test, SCIM	Significant results between groups were found in: mFRT scores (*p* = 0.01); t-shirt test (*p* = 0.01) scores, and in the self-care component of SCIM (*p* = 0.01)
Villiger et al. 2017 [39]	SCIRE: Level 4	IG: Home-based VR-augmented training	4–5 times/week	30–45 min	4 weeks	-Standing balance -Gait -Mobility -Motor function -Functional performance	10mWT, 6minWT; BBS, TUG, LEMS, SCIM, WISCI II, PGIC	Significant differences were found in TUG (*p* = 0.005), BBS (*p* = 0.008), and motor function (*p* = 0.008)
An and Park 2018 [46]	SCIRE: Level 4	IG: IREX video-capture VR system	3 times/week	30 min	6 weeks	-Standing balance -Vertical mobility -Tasks performance	Limits of stability, BBS, TUG, WISCI II, ABC	Significant results were found in: Limits of stability (*p* < 0.01); BBS (*p* < 0.001) TUG (*p* < 0.05), WISCI II (*p* < 0.05), ABC (*p* < 0.05)
van Dijsseldonk et al. 2018 [47]	SCIRE: Level 4	IG: VICON video-capture VR system + treadmill + force plates	2 times/week	60 min	6 weeks	-Standing balance -Walking speed Stabilometric parameters of gait, balance, and mobility	2minWT, ABC, Biomechanical gait stability measures	Significant results were found in walking speed (*p* < 0.001), stride length (*p* < 0.001), stability measures in AP direction (*p* < 0.001)

2minWT: 2-minutes Walking Test; 6minWT: 6 minWalking Test; 10mWT: 10 Meter Walking Test; ABC: Activities-specific Balance Confidence; BBS: Berg Balance Scale; BI: Barthel index; CG: Comparison group; FIM: Functional independence measure; FRT: Functional Reach Test; IG: Intervention group; LEMS: Lower Extremity Motor Score; mFRT: Modified Functional Reach Test, ND: Not described; PGIC: Patients’ Global Impression of Change; SCIM: Spinal cord independence measure; TUG: Timed Up and Go; VR: Virtual reality; WISCI II: Walking Index for Spinal Cord Injury-II.

**Table 3:** PEDro scores obtained by the different studies included in the systematic review.

Study	1	2	3	4	5	6	7	8	9	10	11	Total
D’Addio et al. 2014 [22]	-	Yes	No	No	No	No	No	No	Yes	Yes	Yes	4
Tak et al. 2015 [44]	-	Yes	No	Yes	No	No	Yes	Yes	Yes	Yes	Yes	7
Khurana et al. 2017 [37]	-	Yes	No	Yes	No	Yes	Yes	Yes	Yes	Yes	Yes	8

Range: 0–10. Item 1 is not used in the method score.
